# Supplementary material for: Identification of Freezing-Responsive microRNAs and Their Targets in Chinese Jujube by Small RNA and Degradome Sequencing
Source: Int J Mol Sci. 2026 Sep 4;27(17):7912. doi: 10.3390/ijms27177912 (PMC13566890; doi:10.3390/ijms27177912)
Supplement: Supplementary file 1 [file ijms-27-07912-s001.zip › Supplementary Material S2/novel_miR_65.pdf]

Provisional ID	:	novel_miR_65
Score total	:	0.7
Score for star read(s)	:	-1.3
Score for read counts	:	0
Score for mfe	:	0.4
Score for randfold	:	1.6
Score for cons. seed	:	
Total read count	:	146
Mature read count	:	56
Loop read count	:	90
Star read count	:	0

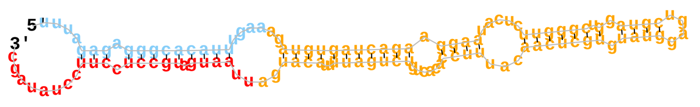

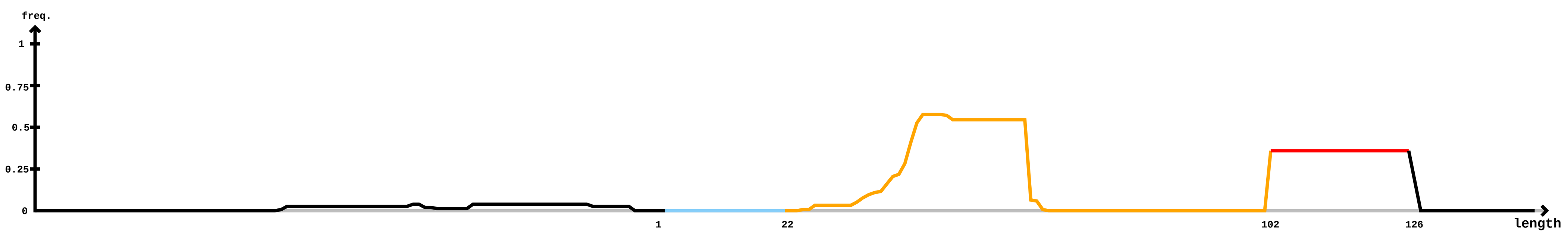


```
                                                                                                             Star                                                                                                     Mature
5'- ucaaucucacuuuagcgaucaacuuguuuuauuccuuucuaggaacugugagauuuaaucuugacccguuuagugagcauagugaagcugaucuuugggaagcuuuagagagggcacauuugaaagaugugaucagaaggaauacucuugggcuggaugcugagguaugugcucaacauuuccacacugucugauuuuacaugauuaaugaugccuccuuccuauagcuuuucauuauuagaagauauua -3'   exp
    .....((((((..((((.((((...((((((((((....))))...)))))))......))))..)))).))))))..(((((...(((((...((((((((.(((.((((((((((......((((((((((.((((.....((((((.(.((((....)))).)))))))...))))......)))))))..))))....)))).)))))...))))).)))))))))..)))))....)))))   reads   mm   sample
    .......................................Caggaacugugagauuuaaucuugac.........................................................................................................................................................................................   1   1   LT-1
    ........................................aggaacugugagauuuaaucuug...........................................................................................................................................................................................   3   0   LT-2
    .............................................................ugacccguuuagugagcauagugaagcuga...............................................................................................................................................................   2   0   JCK-2
    .......................................................................agugagcauagugaagcugaucuuugg........................................................................................................................................................   1   0   JCK-1
    .......................................................................agugagcauagugaagcugaucuuugg........................................................................................................................................................   3   0   JCK-2
    ..............................................................................................................................augugaucagaaggaauacucuug....................................................................................................   1   0   LT-1
    ................................................................................................................................gugaucagaaggaauacucuugg...................................................................................................   3   0   LT-2
    ................................................................................................................................gugaucCgaaggaauacucuugg...................................................................................................   1   1   LT-2
    .......................................................................................................................................gaaggaauacucuugggcuggaugcugag......................................................................................   3   0   LCK-3
    ........................................................................................................................................aaggaauacucuugggcuggaugcugag......................................................................................   4   0   LCK-3
    .........................................................................................................................................aggaauacucuugggcuggaugcugag......................................................................................   2   0   JT-3
    .........................................................................................................................................aggaauacucuugggcuggaugcugaggu....................................................................................   1   0   LT-2
    ..........................................................................................................................................ggaauacucuugggcuggaugcugag......................................................................................   1   0   LT-3
    ..........................................................................................................................................ggaauacucuugggcuggaugcugag......................................................................................   1   0   LT-1
    ...........................................................................................................................................gaauacucuugggcuggaugcugagguC...................................................................................   1   1   JCK-2
    ............................................................................................................................................aauacucuugggcuggaugcugag......................................................................................   2   0   LT-1
    ............................................................................................................................................aauacucuugggcuggaugcugag......................................................................................   1   0   LT-3
    ............................................................................................................................................aauacucuugggcuggaugcugag......................................................................................   3   0   JCK-1
    ............................................................................................................................................aauacucuugggcuggaugcugagg.....................................................................................   1   0   LT-2
    .............................................................................................................................................auacucuugggcuggaugcugaggu....................................................................................   7   0   JCK-2
    ..............................................................................................................................................uUcucuugggcuggaugcugag......................................................................................   2   1   LT-3
    ...............................................................................................................................................acucuugggcuggaugcugag......................................................................................   3   0   LT-1
    ...............................................................................................................................................Ucucuugggcuggaugcugag......................................................................................   4   1   JT-3
    ...............................................................................................................................................Ucucuugggcuggaugcugag......................................................................................   3   1   JT-2
    ................................................................................................................................................cucuugggcuggaugcugag......................................................................................   3   0   LT-2
    ................................................................................................................................................cucuugggcuggaugcugag......................................................................................   1   0   LT-1
    ................................................................................................................................................cNcuugggcuggaugcugag......................................................................................   1   1   JT-1
    ................................................................................................................................................cucuugggcuggaugcugag......................................................................................   14  0   JT-1
    ................................................................................................................................................cucuugggcuggaugcugag......................................................................................   1   0   LT-3
    .................................................................................................................................................ucuugggcuggaugcugag......................................................................................   5   0   LT-2
    .................................................................................................................................................ucuugggcugUaugcugag......................................................................................   1   1   LT-1
    .................................................................................................................................................ucuugggcuggaugcugag......................................................................................   5   0   LCK-3
    .................................................................................................................................................ucuugggcuggaugcugag......................................................................................   3   0   LT-1
    .................................................................................................................................................ucuugggcuggaugcugag......................................................................................   4   0   LT-3
```

```
                                                                                                                           Star                                                                                                 Mature
ucaaucucacuuuagcgaucaacuuguuuuauuccuuucuaggaacugugagauuuaaucuugacccguuuagugagcauagugaagcugaucuuugggaagcuuuagagagggcacauuugaaagaugugaucagaaggaauacucuugggcuggaugcugagguaugugcucaacauuuccacacugucugauuuuacaugauuaaugaugccuccuuccuauagcuuuucauuauuagaagauauua
..................................................................................................................................................cuugggcugUaugcugag......................................................................................   1   1   LCK-3
..................................................................................................................................................cuugggcuggaugcugag......................................................................................   1   0   LT-2
..................................................................................................................................................cuugggcuggaugcugag......................................................................................   2   0   LT-3
..................................................................................................................................................cuugggcuggaugcugag......................................................................................   1   0   JT-2
..................................................................................................................................................cuugggcuggaugcugag......................................................................................   3   0   JT-3
............................................................................................................................................................................................................uuaaugaugccuccuuccuauagc......................   31  0   LCK-2
............................................................................................................................................................................................................uuaaugaugccuccuuccuauagc......................   25  0   LCK-1
```
